# An exploratory review of HIV prevention mass media campaigns targeting men who have sex with men

**DOI:** 10.1186/1471-2458-14-616

**Published:** 2014-06-18

**Authors:** Rebecca S French, Chris Bonell, Kaye Wellings, Peter Weatherburn

**Affiliations:** 1 Department of Social and Environmental Research, London School of Hygiene & Tropical Medicine, 15-17 Tavistock Place, London WC1H 9SH, UK; 2 Sociology and Social Policy, Department of Children, Families and Health, Institute of Education, University of London, 18 Woburn Square, London WC1H 0NR, UK

**Keywords:** Mass media, Men who have sex with men, HIV, Systematic review, Health promotion

## Abstract

**Background:**

Men who have sex with men (MSM) are at increased risk of HIV infection in both high- and low-income settings. Mass media campaigns have been used as a means of communicating HIV health promotion messages to large audiences of MSM. There is no consensus on which designs are most appropriate to evaluate the process and outcomes of such interventions.

**Methods:**

An exploratory review was conducted to assess research examining awareness, acceptability, effects on HIV testing, disclosure and sexual risk, and cost-effectiveness of HIV mass media campaigns targeting MSM. We searched for quantitative and qualitative studies published between 1990 and May 2011 via the Cochrane Central Register of Controlled Trials, MEDLINE, EMBASE, Psych Info, ISI Web of Science, OpenGrey and COPAC, and contacting experts. No exclusions were made on the basis of study design or methods because our primary aim was to map evidence. We appraised study quality and present a narrative synthesis of findings.

**Results:**

Sixteen reports from 12 studies were included. All were from high-income countries and most examined multi-media interventions. Half of the studies were single cross-sectional surveys. Three repeat cross-sectional studies collected data pre and post the campaign launch. The remaining three studies monitored routine data. Three studies included a nested qualitative component. Campaign coverage was the most commonly reported outcome (9 studies). Imagery, tone of language, content and relevance were identified in the qualitative research as factors influencing campaign acceptability. HIV testing rates (or intention to test) were reported by five studies. Two studies reported that testing rates were higher among men who had seen the campaigns compared to men who had not, but this may reflect confounding. Findings were less consistent regarding reductions in sexual risk behaviours (4 studies). None of the studies examined cost-effectiveness.

**Conclusions:**

Campaigns aim to provide MSM with information to help prevent transmission of HIV and to address increasing motivation and changing norms towards precautionary behaviours. However, the limitations of mass media in imparting skills in effecting behaviour change should be recognised, and campaigns supplemented by additional components may be better-suited to achieving these goals.

## Background

Worldwide, sex between men accounts for between 5 and 10% of HIV infections, but the proportion is far higher in much of the developed world where sex between men is the most common means of transmission [1]. Sex between men is also a prominent feature in the epidemiology of HIV in other regions such as Latin America where men who have sex with men (MSM) are at increased risk of HIV infection.

Mass media interventions have the potential to reach large audiences, providing them with information and raising awareness. Campaigns can also present role models and aim to change normative beliefs, as well as helping put health issues on policy-makers’ agenda [2]. Mass media interventions can potentially reach individuals or groups who may not be accessing other statutory or community-based interventions. They may use broadcast media, such as television, radio or film; print media, such as posters and newspapers; outdoor media, such as billboards; or digital media, such as the internet [3].

Although HIV prevention mass media campaigns have been criticised for using weak evaluation design [4,5], there is no consensus on which designs are most appropriate for evaluating process and outcomes [6]. While randomised controlled trials (RCTs) provide the most rigorous means of evaluating public health interventions, they are not generally applicable to mass media interventions because of obvious challenges concerning contamination or the lack of sufficient units for statistical power [2]. Alternatives include interrupted time-series analyses comparing repeat data on outcomes from a population before and after exposure, and cross-sectional studies comparing outcomes among exposed and unexposed individuals. Which of these provide the least biased estimates of effect is likely to depend on context; for example intervention coverage, secular trends in outcomes and likely effect sizes [6]. The evaluation of the effects of mass media interventions on MSM is further complicated by the lack of a sampling frame for this population necessitating convenience samples [7]. Furthermore, other questions of importance to policy-makers such as intervention coverage and acceptability are also not amenable to experimental designs, and in the case of acceptability may be examined via quantitative and qualitative research.

A Cochrane systematic review conducted by Vidanapathirana et al. in 2005 assessed the effects of mass media on HIV testing among the general population and specific target groups, including MSM [8]. The authors concluded mass media campaigns were effective in increasing testing in the general population in the short-term, although no long-term impacts on HIV testing were observed. However, only one of the studies included in the review targeted MSM [9].

Given the lack of previous reviews of HIV prevention mass media interventions targeting MSM, we aimed to examine literature in this area. Given our interest in examining questions of awareness and acceptability as well as effectiveness and cost-effectiveness, and given the lack of consensus on which designs are most appropriate, our exploratory review aimed to systematically map evidence in this area, appraise its quality, and narratively synthesise its findings. The following research questions (RQ) are examined: (RQ1) How successfully do HIV prevention mass media interventions achieve awareness among their target audience(s) of MSM? (RQ2) Does mode of delivery affect campaign awareness among MSM? (3) Are HIV prevention mass media campaigns acceptable to MSM? (RQ4) What influences campaign acceptability to MSM? (RQ5) Are HIV prevention campaigns effective or cost-effective in modifying HIV knowledge or attitudes, reducing sexual risk behaviour, and promoting HIV testing and HIV disclosure among MSM, when compared with pre-intervention or non-exposed participants? (RQ6) In what ways do intervention characteristics appear to influence awareness, acceptability or effectiveness?

## Methods

Our exploratory review was informed by PRISMA guidelines (see Additional file 1) [10]. A protocol was not published but a priori methods were used as described below.

### Search strategy

The following electronic bibliographic databases were searched (from January 1990 to May 2011): the Cochrane Library, MEDLINE, EMBASE, Psych Info and ISI Web of Science. Two databases of grey literature, OpenGrey and COPAC, were searched in December 2012. A search strategy using thesaurus and non-thesaurus terms as appropriate to each database relating to the concepts of MSM, media and HIV, adapted from the strategy used by Vidanapathirana et al. (see Additional file 2) [8]. Relevant websites were also searched, including the World Health Organization, Centers for Disease Control, Diffusion of Effective Behavioral Interventions and the Joint United Nations Programme on HIV/AIDS. The reference lists of related reviews and included articles were searched for additional citations. Authors of included studies and other experts in the field were contacted by email to identify further studies.

### Criteria for selecting studies

Both published and unpublished literature was included. Included reports met the following criteria:

Published in English between 1990 and May 2011.

#### Target population

Any study where MSM were an intervention’s target group, irrespective of sexual identity. This included interventions that solely targeted MSM or where interventions also targeted other groups but study results were reported for MSM as a subgroup. Evaluations where it was not possible to disaggregate the intervention’s awareness, effectiveness or acceptability amongst MSM from other target groups were excluded. Studies of campaigns targeting health professionals were excluded.

#### Intervention

Mass media campaigns relating to HIV health promotion that targeted MSM were included. Unpaid for media coverage and interactive media health promotion interventions (such as use of internet chat rooms) were excluded. Interventions that only included small media, such as leaflets, were excluded, but those where mass media were complemented with small media were included. Outcome evaluations of complex programme interventions which included both a mass media component and non-media components were excluded, as were mass media campaigns relating solely to other aspects of sexual health. Laboratory studies which artificially exposed a research sample to an intervention were also excluded.

#### Comparators

Pre-intervention or non-exposed study participants.

#### Study design

As explained above, our review sought to map and appraise a variety of study designs. Therefore, in relation to each of our research questions, studies were not excluded on the basis of their design. Instead, our appraisal systematically assessed the potential internal and external validity of studies (see below).

Descriptions of mass media campaigns with no form of evaluation and studies limited to piloting or pre-testing were excluded.

#### Outcomes

Evaluations were included which examined at least one of the following outcomes at any post-intervention time-point: HIV knowledge or attitudes, HIV testing, HIV disclosure and sexual risk behaviours. No limitations were put on length of follow-up.

References identified through our search were downloaded into an Excel (Microsoft 2010) spreadsheet. Titles and abstracts were screened by one reviewer. Full texts were obtained for review when titles and abstracts met our inclusion criteria or when there was any ambiguity about the decision for inclusion. These were screened by one reviewer, using a screening sheet detailing inclusion and exclusion criteria, and checked by a second with no disagreements occurring.

### Data extraction and quality assessment

Data on intervention (media employed, aims, theory of change, any initial formative research or piloting, setting, target population) and study (aims, design, sampling, response rates, data collection, analysis) was extracted from studies by one reviewer and checked by another with any disagreements being resolved by discussion. Data extraction forms had been piloted on two studies.

Quality assessment was conducted at the study level using tools developed specifically for this review. Existing Cochrane [11] and TREND [12] (Transparent Reporting of Evaluations with Non-Randomized Designs) could not be used because these focus respectively on RCTs and on non-randomized studies with external control groups, whereas, as discussed above, our evaluations used a range of designs to examine multiple questions with no consensus in the field as to which are the most rigorous. Quantitative studies were assessed in terms of mimimizing confounding, selection and information bias, reverse causality and random error. Qualitative studies were assessed using established criteria [13] addressing sampling, data collection, data analysis, the extent to which the study findings are grounded in the data, whether the study privileges the perspectives of participants, and the breadth and depth of findings. All reports were quality assessed by one reviewer and checked by another with any differences resolved by discussion.

### Synthesis of findings

Given the exploratory aims of this review and the lack of homogeneity in study design and aims, measures and interventions, it was not appropriate to undertake meta-analysis and narrative synthesis was instead undertaken, using similar approaches to those used in previous well-conducted reviews.

Given the small number of qualitative studies found in this review and the lack of overlap in the substantive topics addressed, the decision was taken not to attempt a systematic synthesis, for example via meta-ethnography, and instead to limit reporting to presentation of findings and conclusions of the studies on their own terms.

## Results

**Figure 1:**
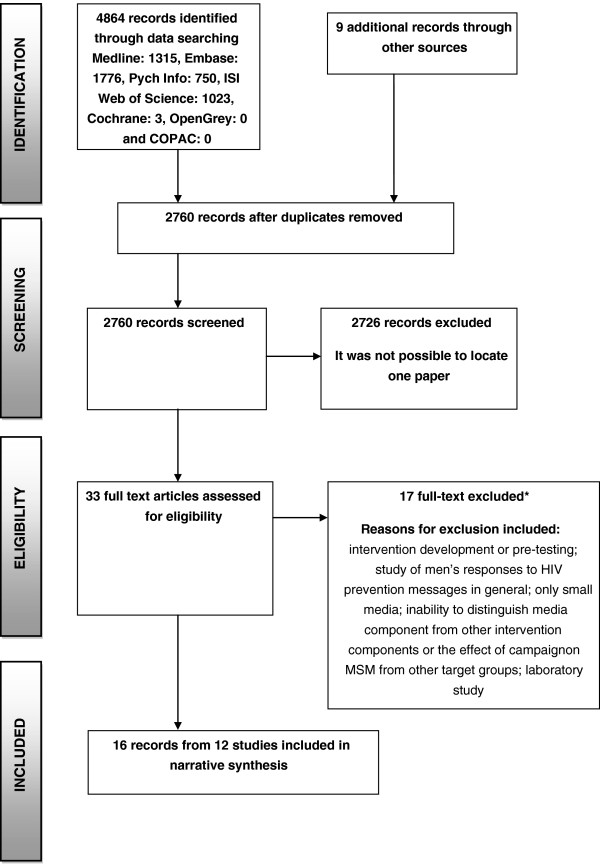
PRISMA flow diagram.

The database search identified 2751 reports. After preliminary screening of titles and abstracts, 25 reports were examined in full (Figure 1). Seven studies met the inclusion criteria [9,14-19]; 17 reports were excluded; one of these because it could not be located [20]. A further nine reports from five additional studies, were identified through website searches, reference list searches and contact with experts [21-29]. Therefore, in total, 16 reports from 12 studies were included.

## Study focus

Nine studies examined campaign awareness [14-18,21-28]. Seven assessed acceptability or influences on acceptability [15,16,18,21-28]. None assessed effects on knowledge or attitude outcomes; five examined effects on HIV testing [9,16,19,21] or intention to test [22]; and four examined effects on sexual behaviour, including unprotected anal sexual intercourse [9], condom use [16,29], median number of sexual partners [29] and changes in sexual practice [15,28]. No studies examined cost-effectiveness.

### Study context and target population

Seven of the identified studies were conducted in the UK [9,17,19,21,23-27,29], four in the USA [14,16,18,22], and one in Canada [15,28].

Seven interventions targeted all MSM [16-19,21,23-26,29]. The remainder targeted specific sub-groups of MSM by age [9,27] or ethnic sub-groups [9], recent unprotected sexual intercourse with men of unknown or discordant HIV status [15,22,28] and perceived HIV-negative serostatus [14]. None of the interventions reported aiming to target MSM according to sexual identity.

### Interventions

Details of interventions are provided in Table 1. Most adopted a multi-media approach, with some of these including the internet [15,16,21-28]. Only one included television and radio along with other media [19]. Seven multi-media interventions included small media, such as leaflets and “knick-knacks” (small branded novelty items such as key-rings, condom packs, and sweets), to complement mass media campaign [9,15,18,21-28].

**Table 1:** Intervention characteristics

**Study**	**Intervention**	**Theoretical framework**	**Formative work**	**Setting**	**Location of delivery**	**Population**
Hecht et al. [14]	“Are you Iffy?” a social marketing campaign to encourage MSM to reassess their HIV-negative status. 140 posters, 7 billboards and 10 newspaper ads	Health belief Model	Focus groups and community field-testing	San Francisco, USA	Bars/clubs, toilets, subways, bus terminals and newspaper kiosks	HIV negative MSM
Hilliam et al. [21]*	“HIV Wake-Up Campaign” to provide information on HIV, benefits of prevention and regular testing and where to get further advice. Posters, web adverts and dedicated website, complemented with small print media, i.e. leaflets	Not stated	Not stated	Scotland	Internet	MSM and non-HIV specialist professionals
					Not stated where print media distributed	
Sigma Research [23-26]*	16 mass media adverts placed in press, on websites and as posters, complemented with small media, such knik-knaks	Not stated	2007: Themes for interventions identified in meetings with partners. Pre-testing with focus groups.	UK	National and regional press and where appropriate national HIV positive press. Posters in gay bars, saunas and clubs	MSM
	Adverts and their aims (number of images, launch date, display costs):					
	*Assume nothing* (4 images, July 1997, £73,000)				London underground	
	*Think, Talk, Time Test* (3 images, Jan. 1998, £67,000)					
	*What am I?.. See to it. (1* (1 image, July 1998, £70,000)					
	*What on Your Mind?* (1 image, Jan. 1999, £70,000)					
	*Homophobia* – (1 image in gay media, Oct 1999; £4,934; 3 images in general media, Aug 1999, £74,616)					
	*Better of knowing* – (5 images, Feb 2000; £52,254)					
	*Facts for life –* to provide information on HIV risks (9 images, Sept 2000, £39, 808; Jan 2003)					
	*In two minds -* to illustrate dilemmas between “thoughts connected to the head (relating to risk reduction) and with the cock/crotch (less rational…)” (10 images, Nov. 2000; £31,114)					
	*Just unbelievable* - to highlight the presumption that all HIV positive partners will disclose their status (3 images, Oct 2001, £20, 252)					
	*Clever Dick -* to promote condom use (5 images, March 2002, £22,046)					
	*Biology of transmission* – to increase awareness of rectum’s and anus’s fragility and absorbency (3 images, Oct 2002, £20,661)					
	*Think again* – to show divergent thoughts and concerns about HIV transmission and exposure (6 images, Nov 2003, £20, 326)					
	*Infection situations* – to illustrate possible adverse outcomes associated with sexual risks (5 images, April 2004, £24, 191)					
	*Be confident, be covered* – to promote use of condoms (3 images, Feb 2005 £16,923)					
	*PEP* – to increase knowledge of post-exposure prophylaxis (PEP) and is availability after sexual exposure to HIV (1 image. June 2004 and July 2005, £9, 428)					
	*Closer* – to increase awareness of local and national HIV prevalence and to get men to reconsider their HIV risk (6 images, Jan 2006, cost not reported)					
Roedling et al. [29]	A campaign to provide MSM with information about PEP and where it is available	Not stated	Not stated	London and Brighton, UK	Adverts placed in gay press and ‘other material’	MSM
Hartfield et al. [22]*	“It’s the Little Prick You Can Deal With” campaign to encourage MSM at high risk of HIV to have HIV test every 3 months. Gay-orientated websites, billboards, pavement chalk drawings, complemented with small print media and knick-knacks (e.g. coasters)	Not stated	Pre-testing with community members	Seattle, USA	Outside and inside gay bars and saunas	MSM at high risk of HIV infection, defined as those who have had unprotected sexual intercourse with a partner of unknown or discordant HIV status in last year
					Internet	
Katzman et al. [16]	“Community Manifesto” to identify sexual health issues and to promote positive sexual health for MSM	Not stated	Task force meetings with involvement from health agencies and community members	Seattle & King County, USA	Placed in 2 weekly Seattle papers, freely distributed. Also posted on a gay health web site in English and Spanish	MSM
Lombardo & Léger [15,28]*	“Think Again” campaign (adapted from the US “Assumptions” campaign) to encourage men to challenge assumptions around partners’ HIV status. Ultimate goal to reduce unprotected anal intercourse between men of discordant HIV status and thereby reduce HIV incidence. Multi-media complemented with small print media and knick-knacks (e.g. coasters). Cost of campaign = $250,000	Used social marketing concepts	9 focus groups (47 participants)	Canada	National campaign - billboards, gay venues and Internet	HIV positive and HIV negative men having unprotected sexual intercourse with men whose HIV status is unknown to them
The TASC Agency [27]*	“Equal” campaign to promote safer sex, condom and lube use, and regular sexual health check ups. Posters	Not mentioned	Not mentioned	Scotland	Not mentioned	MSM aged 25-40 years
McOwan et al. [9]	HIV testing campaign ‘gimme 5 minutes’ which ran between March-May 2000. ‘Peer’ images (different photos representing each of the target groups) with same accompanying text covering topics relating to pre-test discussions, making decision to test and information about testing services at the campaign clinic. Newspaper adverts and posers, complemented with small print media. Cost around £10,000	Not mentioned	Not mentioned	London, UK	Full page advertisements in a free tabloid newspaper, 100 posters in Central London bars	MSM, particularly targeting men of Black and Southern European origin and men under 25-years-old
Sherr et al. [17]	“Try this HIV test” campaign to encourage homosexual men to consider having an HIV test in light of recent advances in HIV treatments.	Not mentioned	Not mentioned	London, UK	Gay press	MSM
Dawson & Hartfield [18]	Newspaper comic strip “Stella Seattle” “to clarify information about controversial transmission issues and to encourage HIV testing”	Not mentioned	Mention formative research was undertaken and focus groups set up to test first few comic strips were tested	Seattle, USA	Comic strip ran weekly for 4 months in 2 local newspapers with a large gay readership	MSM
	Campaign began Aug 1993. Cost $9,500 (included media placement of 15-episode strip, artist fees and staff time)					
Griffith et al. [19]	Statutory HIV educational campaigns, including television and radio. Early campaigns were general and latter ones were targeted to specific subpopulations, including gay and bisexual men. Of the 38 media interventions 6 aimed at gay men and 2 at bisexual men	Numerous media campaigns	Not mentioned	London, UK	Across TV, radio and gay press	Gay and bisexual men
	Multi-media					

*‘Grey’ literature.

Across all interventions, posters were mainly placed in gay commercial venues and other gay community settings. Other studies described placing posters in other settings, such as around transport hubs [14,15,28].

Formative research was used to inform the development of the campaign concepts, content and materials in five interventions, including: use of focus groups with the target population [14,15,18,23-26,28], meetings with health agencies or community members [16,23-26], and community field testing [14,22]. This formative work helped ensure that campaign content addressed men’s preferences and needs, and as described in the study by Lombardo & Léger, it also helped to adapt an existing intervention to a new geographical setting while maintaining message consistency [15,28].

Most interventions aimed to provide information on HIV prevention strategies and encourage HIV testing (Table 1). While most of the campaigns had moved away from simple ‘use a condom’ messages, few aimed to provide men with information on negotiating safer sex or disclosing their HIV status to a sexual partner. Furthermore, only two studies described the theory of change underlying the intervention; one drawing on the Health Belief Model [14] and the other on social marketing concepts [15,28].

Campaign costs were reported in four studies [9,15,18,23-26,28]. Costs ranged from $250,000 for the national campaign in Canada [15,28] to £9,500 for media placement, artists’ fees and staff time for the “Stella Seattle” newspaper comic strip [18].

### Study design and quality

The 12 studies from which our 16 reports drew included a variety of designs; none were solely qualitative studies (Table 2). Three studies used pre- and post-test repeat cross-sectional designs [14,21,22]. Post-test surveys were conducted between 0-5 months after the campaign launch. Two of these studies also examined outcomes in the post-test survey according to individuals’ exposure to the campaign [21,22], while the other examined testing before and after the campaign irrespective of individual exposure [14]. A further six studies involved a single post-intervention cross-sectional survey [15-18,23-28]. Two other studies drew on routine data to examine HIV testing before and after the intervention [19,29]. Another study examined an intervention to increase HIV testing in one sexual health clinic compared with two non-randomized control clinics, collecting data on HIV testing retrospectively [9].

**Table 2:** Study characteristics

**Study**	**Aims**	**Design**	**Sample**	**Study process and outcome measures**	**Results**
Hecht et al. [14]	To compare certainty of HIV negative status before and after the campaign	Repeat pre-test/post-test cross-sectional study (campaign May - July 2008, post-test July - Oct 2008). Part of National HIV Behavioral Surveillance	Recruitment: MSM venue-based, time-space sampling	Campaign awareness	45% reported they had seen the campaign. Of these:
			Response rate: Not reported		64% correctly identified the subject of the campaign
			Sample size: 316 men		
			Analysis confined to 255 men who reported being HIV negative		
Hilliam et al. [21]*	To measure campaign awareness; HIV awareness; attitudes toward testing, prevention and safe sex; and behaviour change	Repeat pre-test/post-test cross-sectional study - post-test 4-5 months after launch	Recruitment: Via LGBT and HIV organisation websites, and Gaydar (post-test only). Men recruited via Gaydar (G) were analysed separately from those recruited via other non-Gaydar (nG) websites due to differences observed between samples	Campaign awareness	3-13% reported non-prompted awareness of campaign
			Response rate: Not reported		69%^nG^ and 82%^G^ reported prompted awareness of campaign (men most commonly reported seeing the web adverts 50%^nG^ and 77%^G^, followed by the web site 17%^G^ and 29%^nG^, and finally by the posters 27%^G^ and 32%^nG^)
			Sample size: Pre-test sample = 88, Post-test sample = 775	Campaign attributes	Campaign attributes most commonly reported by men: clear message (62%^nG^ and 63%^G^) and relevance (51% ^G^ and 61%^nG^). Campaign attributes least commonly reported by men: motivating (11%^nG^ and 20%^G^) and trustworthy (23%^nG^ and 25%^G^)
				HIV test	Men who had seen the campaign were more likely to report having had an HIV test in the last 6 months than those who had not seen the campaign, 33%^G^ and 38^nG^% versus 9%^nG^ and 16%^G^, respectively
Sigma Research [23-26]*		Repeat cross-sectional surveys – Gay Men’s Sex Survey (GMSS) questionnaire	Recruitment: Via Pride-type events across UK and the Internet	Campaign Awareness	2005
		Focus groups and interviews	A variety of recruitment methods described for nested qualitative component, including established agency networks, e-newsletters, fliers and use of snowballing techniques. Purposive sampling, UK cities		*Be confident, be covered*: 32% recognised, and of those 52.4% had read
			Response rate: Not reported		*PEP*: 16.1% recognised, and of those 56.6% had read
			Sample size:		2004
			Surveys		*Infection situations*: 18.6% recognised, and of those 51.6% had read
			Between 1997-2000 data gathered via face data collection at Pride events, then from 2001 via booklets and online		*Think again*: 29.1% recognised, and of those 54.0% had read
			2005: N = 12,322		2003
			2004: N = 11,909		*Biology of transmission*: 26.1% recognised, and of those 50.3% had read
			2003: N = 9,482		2002
			2002 N = 11,046 (booklet = 3515, online = 7531)		*Just as unbelievable*: 30.7% recognised (40.4% of booklet users and 26.5% online users), and of those that recognised 64.6% (booklet) and 58.2% (online) had read
			2001 N = 9226 (Pride attendees = 2401, booklet = 2384, web = 4441)		*Clever dick*: 31.9% recognised (43.9% of booklet users and 26.7% online users), and of those that recognised 72.2% (booklet) and 64.8% (online) had read
			2000 N = 312		2001
			1999 N = 313		*Facts for life:* Recognised by 42.1% of Pride attendees, 43.0% of those using the booklet and 24.8% of those online, and of those that recognised 54.5% (booklet) and 41.4% (online) had read
			1998 N = 294		*In two minds?* Recognised by 62.9% of Pride attendees, 59.6% of those using the booklet and 39.3% of those online, and of those that recognised 71.5% (booklet) and 63.1% (online) had read
			Focus groups		2000
			2009: 6 groups, 49 men		*Better off knowing*: 48.1% recognised
			2004: 7 groups, 33 men		*What’s on Your Mind*: 31.7% recognised
			2003: 5 groups, 46 men		*What am I?.. See to it:* 47.1% recognised
			2001: 5 groups, 37 men		1999
			Interviews		*Homophobia:* 34.1% recognised image in gay press, 36.5% recognised images in general media
			2000: 68		*What’s on Your Mind*: 54.3% recognised
			1998: 71		*What am I?.. See to it:* 47.9% recognised
			1997: 62		*Think, Talk, Time to Test:* 35.1% recognised
					1998
					*Think, Talk, Time to Test:* 40.5% recognised, and of those 62.5% had read
					*Assume nothing:* 44.6% recognised, and of those 56.1% had read
Roedling et al. [29]	To compare clinical data, exposure characteristics, follow-up and awareness of post-exposure prophylaxis following sexual exposure to HIV pre and post campaign	Retrospective case note review from 2004 pre and post campaign	Recruitment: Not applicable. Case notes for all those attending for PEP in 2004 included	Sexual behaviour	Condom use
		Campaign launched in July 2008	Response rate: not applicable		Pre-campaign 20/33 (61%)
			Sample size: 216 attendees requested PEP, data available on 197 (91%)		Post-campaign 39/66 (59%), p = 1.00
			Analysis: Confined to 112 MSM commencing PEPSE, pre-campaign n = 36 and post-campaign n = 76		Median number of sexual partners in the previous 3 months
					Pre-campaign 3 (range 1-50)
					Post-campaign 4 (1-100), p = 0.51
Hartfield et al. [22]*	To evaluate campaign coverage and impact	Repeat pre-test/post-test cross-sectional study. Campaign launched June-Aug 2008. Pre (March – May 2008) and post campaign (June-Dec. 2008) survey	Recruitment: Via MSM venues	Campaign awareness	75% reported exposure (24% unaided and a further 50% prompted)
			Response rate: Not reported	Campaign acceptability	80% of those who saw campaign very positive/positive. Only 3% negative
			Sample size: Baseline survey n = 197	Intention to have HIV test	38% of those who had seen the campaign and were HIV negative (n = 279) said they would test more frequently due to the campaign
			Post-campaign survey n = 464		
Katzman et al. [16]	To examine the potential impact of the manifesto	Cross-sectional survey and focus groups (in English and Spanish)	Recruitment: Survey posted on web, left in 38 gay venues for mail-in and distributed by street intercepts. Method of focus group recruitment not stated	Campaign awareness	84% seen or heard manifesto
			Response rate: 2506 surveys distributed in gay venues and 137 surveys returned (5.5%). 69 surveys from women and men without a partner excluded	Campaign acceptability	Of those who had seen it: 61% strongly agreed/agreed with the manifesto, 19% disagreed/strongly disagreed
			Sample size: Survey n = 103	HIV testing	13% had HIV test
			9 Focus groups (139 participants) with representation of gay men both HIV positive and negative	HIV status disclosure	12% disclosed HIV status 10% asked partner to disclose status
				Sexual behaviour	16% increased condom use
Lombardo & Léger [15,28]*	To assess impact	Cross-sectional survey	Recruitment: Via “gay spaces” across Canada	Campaign awareness	79% average national exposure Top messages received “rethinks risks” 47%, “protect self and partner” 37% and “use condoms”35%
			Response rate: Not reported	Campaign acceptability	73% found messages appealing
			Sample size: N = 417	Sexual behaviour	48% report message prompted them to change “something” about sexual practices, but men were not asked about the direction of change
The TASC Agency [27]*	Not stated	Cross sectional surveys (2006) and online survey (2007), focus groups and in-depth interviews	Recruitment: For surveys via Internet and a Pride event in Glasgow. Not stated for focus groups and interviews	Campaign awareness	Survey 2006: 82% had seen phase 1 and 2 poster images. 5.3% had visited the Equal website
			Response rate: Not reported		Online survey: 8/26 had previously seen the Phase 1 posters; 9/24 the Phase 2 posters and 15/25 the Phase 3 ones
			Sample size: Survey 2006: 222 men responded. Analysis confined to men aged 25-40 years, N = 116	Campaign acceptability	Survey 2006: Phase 1 posters 57% reported that they “Love’em” or “They’re good”; Phase 2 posters 53%
			Online survey: N = 27		
			6 focus groups with 28 participants		
			10 interviews		
McOwan et al. [9]	To evaluate effect of an HIV testing campaign	Retrospective case note review of GUM attendees – comparing the same time points across two years and comparing campaign clinic with two other sexual health clinics	Recruitment: Not applicable. Those testing in the three clinics were retrospectively identified through a central laboratory	HIV testing	In the campaign clinic 4.5 fold increase in numbers of men testing in 2000, n = 292, compared to 1999, n = 65, (p < 0.001), 14.0 fold increase in men of Southern European origin (n = 42 in 2000 vs. n = 3 in 1999, p < 0.001), 6.5 increase in Black men (n = 13 in 2000 vs. n = 2 in 1999, p = 0.003) and 9.5 increase in men under 25 (n = 57 in 2000 vs. n = 6 in 1999, p < 0.001)
			Response rate: Not applicable		No significant differences for these outcomes observed in two control clinics. Total number of men testing in 2000 = 236 and in 1999 = 239 (p = 0.982), Southern European men testing n = 37 and 25, respectively (p = 0.341), Black men testing n = 3 and 5, respectively (p = 0.864) and men aged less than 25 years n = 32 and 36, respectively (p = 0.807)
			Sample size: See Results	Sexual behaviour	Unprotected anal intercourse (campaign clinic only)
					Pre campaign 35/65 (53.8%, 95% CI 41.0 - 66.3%)
					Post campaign 156/292 (53.4%, 95% CI 47.5-59.1%)
Sherr et al. [17]	To evaluate permeation (picture recognition), recall of message, endorsement, and decision to have a test	Cross sectional survey	Recruitment: Two sexual health clinics	Campaign awareness	80.1% reported seeing the campaign pictures
			Clinics attendees having an HIV test. In one clinic this included heterosexual men and women, and homosexual men requesting an HIV test and in the other all homosexual men	Decision to have HIV test	25.5% recalled the message (half of this group had correct recall)
			Response rate: Not reported		9.3% reported campaign played important part in decision to have a test
			Sample size:		
			667 individuals completed questionnaire, of these 339 reported they were homosexual or bisexual		
Dawson & Hartfield [18]	To look at exposure to and satisfaction with campaign	Repeat cross sectional surveys	Recruitment: Gay bars and a Pride event at different points in time	Campaign awareness	1993 interviews – 73% familiar with comic
		Structured-interviews	Response rate: Not reported		1993 clinic forms - 32% gay and bisexual clients who tested reported seeing the campaign
		Review of clinic HIV testing and voice mail calls	Sample size:		1994 bar survey – 47% had seen comic strip
			Oct 1993 gay bar structured interviews - number unknown		1994 rally survey – 44% had seen comic strip
			July-Nov 1993 clinic review – number of case notes reviewed not reported	Campaign acceptability	1993 interviews - Of those familiar, 89% positive. Negative comments mainly around format, such as confusing and hard to read
			May 1994 gay bar survey, n = 662		1994 rally survey – Of those who had seen it, 57% liked a lot and 41% thought OK
			June 1994 Gay Pride Rally survey, n = 198		Voice mail – weekly average of 200-400 calls. Report that most were positive
Griffith et al. [19]	To describe association between HIV educational campaigns and long-term testing trends between Sept 1985 – Sept. 1993. Of the 38 media interventions, 6 aimed at gay men and 2 at bisexual men	Continuous – prospective collection of demographic and behavioural data from all GUM attendees having an HIV test	Recruitment: Not applicable	HIV testing	Unable to extract data - trends in testing amongst homosexual and bisexual men annotated with media campaigns shown graphically. Authors report periods of peak testing generally corresponded temporally with increased media coverage
			Response rate: Not applicable		
			Sample size:		
			19 242 tested in three London sexual health clinics, UK		
			12 183 men (37.6% homosexual and 7.9% bisexual)		

LGBT Lesbian, Gay, Bisexual and Transgender.

nG = Non-Gaydar and G = Gaydar. A variety of MSM-related websites were used to recruit men for the survey. However in the post-test Gaydar added. Noted there were differences in reported partnership status and number of partners between Gaydar and non-Gaydar recruited participants, so results presented separately.

*‘Grey’ literature.

Limited information was provided on recruitment and sampling methods. As expected, none of the studies recruited men using random probability sampling. The study by Lombardo & Léger reported that men were recruited at random from convenience samples in ‘gay spaces’ rather than using representative sampling frames [15,28]. Other studies reported using convenience sampling to identify men in gay venues, such as bars and clubs, or locations where gay men were likely to congregate, such as on streets or in parks [14-16,18,22]. Three studies described recruitment via the Internet [16,21,23-26], five accessed GUM attendees [9,17-19,29], and three recruited men at gay community events, such as Pride [18,23-27]. The study by Katzman et al. was the only one to provide information on response rates and reported a low response rate for surveys distributed at gay venues [16]. Thus, the potential for selection bias is strong resulting in over-estimates of coverage and effects. These limitations also suggest the need for caution regarding the external validity of study findings. Information bias is also likely with many of the studies. Men completing questionnaires face-to-face with an interviewer may have been more likely to provide socially desirable responses compared to men self-completing questionnaires, thereby inflating estimates of study coverage, acceptability and effects. Several studies relied on participants’ own attributions to assess intervention effects [9,21,22], and these are likely to be vulnerable to information bias.

Confounding was another major source of potential bias. None of the included studies described attempts to control for confounding either via matching or adjustment of comparison groups. The cross–sectional studies were also vulnerable to potential reverse causality [15-18,23-28].

Three studies included a nested qualitative component [16,23-27]. None of these studies aimed to build theory. One reported methods of recruitment [23-26]. Men were purposively sampled to ensure, for example, representation of different ages and HIV statuses. None of the studies reported on methods used to analyse data. Nor did the studies provide in-depth quotes and thick description of context or aim to build theory.

### Study findings

Findings are presented in Table 2.

#### RQs 1 and 2: Campaign awareness and how this was affected by mode of delivery

Campaign awareness was examined in nine studies [14-18,21-28]. A variety of designs were used to address the question of campaign awareness: four single cross-sectional studies [15-17,27,28] and five repeat cross-sectional studies [14,18,21-26]. Coverage ranged from 3% to 84%, but this is partly an artefact of different methods of elicitation, such as whether prompted or unprompted and the different time-frames used. Recognition of campaign imagery was in every study more prevalent than recall of campaign messages [14,17,23-26,28].

Differences were noted in the characteristics of men who reported campaign awareness compared to those who did not in two studies. Sigma Research observed across surveys that campaigns were more likely to be seen by gay rather than bisexual-identified men, those with more male partners and those who had tested HIV positive compared to those who had not [23-26]. Men under 20 years of age and those over 50 were less likely to recall campaigns compared with men aged 20-50 years, as were those who had not tested for HIV, and those with low educational attainment. Hilliam et al. reported that non-gay identified men were less likely than gay-identified men to report awareness of the campaign when prompted (69% versus 82%, respectively) [21].

Due to heterogeneity of interventions and methods, it was impossible to determine whether or how mode of delivery affected campaign awareness.

#### RQs 3 and 4 Campaign acceptability and influences on this

Seven studies reported on acceptability or on the attributes that may affect acceptability [15,16,18,21-28]. These drew on single cross-sectional [15,16,27,28] and pre/post test cross-sectional design [18,22], as well as focus groups [16,23-26]. Hilliam et al. reported that in their evaluation of the “HIV Wake-up campaign” men were most likely to agree the campaign had a clear message (around a third of men) but least likely to agree that the campaign was motivating (less than 20% of men) [21]. Four themes relating to increased acceptability were evident from the focus group studies: imagery (such as the use of models representative of the gay community, the benefits of comics for explicit material, and the importance of ensuring imagery and the campaign message complement one another); content (such as ensuring messages are not too complex); tone (such as not being patronising or blaming); and relevance (such as making certain messages are appropriate to the target audience) [16,23-27]. The included studies looked at overall acceptability of the campaign rather than the acceptability of different modes of delivery.

#### RQs 5 and 6 Campaign effectiveness and cost-effectiveness, and effect of intervention characteristics on these

##### Knowledge and attitudes

None of the included studies reported on knowledge or attitude outcomes.

#### HIV testing and HIV status disclosure

Five studies reported on HIV testing [9,16,19,21] or intention to test [22]. Two of these examined campaigns where the primary aim was to encourage HIV testing [9,22]. A repeat cross-sectional study with data on HIV testing behaviour pre- and post-intervention reported that testing increased significantly post-intervention, from 16% to 33% among gay-identified men and from 9% to 38% in non-gay-identified men [21]. Another study found that 38% of HIV-negative men reported that they would test more frequently as a result of the intervention [22]. However, another study reported that only 9.3% of men attending a sexual health clinic identified a campaign to encourage HIV testing as an important factor in their decision to have a test, although HIV testing data are not presented [17]. In the study comparing intervention and control clinics [9], increases in HIV testing were observed among MSM in general in the campaign clinic, but the greatest increases were observed among Black, southern European and young men, images of whom featured prominently in the campaign. The authors report that whereas in the year prior to the campaign only one of the 65 MSM testing reported that they did so as a result of an advertisement, 162/292 did so during the campaign. No similar increases in testing were observed in the two comparison clinics. However, these findings should be interpreted with caution: men in the control clinics were not asked about the reasons for testing; other confounding factors could have affected the observed increases in testing and there may have been contamination across clinics. A limitation of the two studies in sexual health clinics was that the samples only included individuals having an HIV test [9,19].

HIV status disclosure was examined in one cross-sectional study, which observed that 12% of men who reported seeing the campaign reported disclosing their HIV status to a partner [16]. However, as there is no comparison group it is difficult to attribute disclosure of HIV status to the campaign.

#### Sexual behaviour outcomes

Four studies reported sexual behaviour outcomes: unprotected anal sexual intercourse [9], condom use [16,29], number of sexual partners [29], and change in sexual practice [15,28]. Two of the studies were single cross-sectional studies asking men about sexual behaviour post-intervention [15,16,28]. The remaining two studies compared pre- and post-intervention measures [9,29], with neither study observing any significant differences. However, both these studies were retrospective case note reviews and are therefore limited by what information was recorded in the case notes.

#### Cost-effectiveness

None of the included studies examined cost-effectiveness of the campaigns.

Due to the broad range of interventions, designs and outcomes, it was not possible to examine how intervention characteristics affected outcomes.

## Discussion

### Summary of key findings

The studies included examined different aspects of intervention process and diverse outcomes. Key limitations in methodology included unrepresentative samples, information bias and lack of control of confounding. We note particular problems with the cross-sectional studies comparing post-intervention measures of exposure, process and outcomes, in that these were likely to be subject to strong confounding and information bias [15-18,23-28]. Repeat interrupted times series, a design used by three of the included studies [14,21,22], are likely to provide less biased estimates of intervention effects.

Intervention awareness was variable and recall of key messages among exposed men was generally poor. There was some evidence of lower awareness among non-gay than gay-identified men. Campaign acceptability was variable, and there was some evidence that attention to imagery, content, tone and campaign relevance could enhance acceptability. We found little rigorous evidence of significant effects of mass media interventions on MSM. There was some evidence of short-term increases in HIV testing. Our exploratory review found no rigorous evidence of intervention effects on sexual behaviour outcomes and on HIV status disclosure. As none of the studies were conducted beyond six months, it was not possible to assess sustained impact on behavioural outcomes. Changes in knowledge or attitudes or cost-effectiveness of the campaigns were not reported by any of the studies. We found no evidence addressing how intervention characteristics might influence effectiveness.

### Limitations

Because of our multiple research questions and the lack of consensus of which designs are most appropriate to examining these, we undertook an exploratory systematic mapping and appraisal of studies of HIV prevention mass media interventions targeting MSM. In the section below, we reflect on the potential for future systematic reviews of mass media interventions to define more focused questions and inclusion criteria for studies. Other limitations should be noted when interpreting our findings. First, most of the published evidence has come from high income countries. Second, only English language papers were included in the review, so literature in other languages would have been missed. Third, although we searched for unpublished literature in multiple ways, those reports we found came mostly from the UK, which may reflect our stronger domestic networks. Unpublished reports which were older or came from other settings are more likely to have been missed, especially perhaps if they reported null or negative findings. Finally, although interventions that only included small media were excluded, some of the multi-media campaigns did include small media, and it is possible that these may have affected outcomes of interest.

### Implications for research

We highlight above the particular weakness of the included studies which drew on post-intervention measures of intervention exposure and outcomes in order to assess intervention effects. Even had these studies attempted to control for confounding differences between those reporting exposure to interventions and those not doing so, substantial residual confounding would very likely have remained because of the subtle differences between those recalling and not recalling campaigns. Therefore, we conclude that any future systematic reviews of mass media should focus on interrupted time-series studies examining pre- and post-intervention measures, drawing either on longitudinal data or from repeat cross-sectional data where this involved consistent sampling methods. Although such studies are vulnerable to confounding by secular trends in the outcomes in question, we conclude that this is a less important source of bias. We therefore also recommend that primary evaluations of mass media effects adopt this design. Nonetheless we stress the importance of cross-sectional studies in assessing awareness and a combination of cross-sectional studies and qualitative research in assessing acceptability.

Our review did not include any studies using interactive media such as smart phone applications or website-based risk assessment tools. Very few studies of such media have yet been published. The use of new technologies warrants further investment and research [30].

Evaluation surveys for the most part found that campaigns were acceptable to MSM. Campaigns need to be relevant to the target audience’s needs and formative work during development with target (and non-target groups if material might be viewed outside gay venues) was seen as key to ensuring imagery, language, tone and content were acceptable. Piloting and pre-testing of campaigns should be considered a prerequisite to any campaign launch.

### Implications for policy

Mass media interventions have the potential to reach large audiences, and their cost is low per individual reached; though it should be noted that most of the costs provided in the studies focused on display and distribution only, and not on staff time and other development costs. We found insufficient evidence to determine whether mass media interventions represent an effective or cost-effective strategy in the prevention of HIV infection amongst MSM. We recommend that further research is required to investigate this, drawing on interrupted time-series designs and focusing on new/interactive media in addition to traditional/static media.

## Conclusion

The aims of the studies in our exploratory review were generally focused on behaviour change, such as HIV disclosure or HIV testing, rather than information provision. A previous systematic review concluded that effective behaviour change interventions require a focus on interpersonal skills development rather than merely the provision of knowledge [31]. The strength of mass media interventions is that they may have a small influence, but on a relatively large portion of the target population. They can also signpost more in-depth interventions, such as one-to-one interventions that are better at addressing motivation and skills. They can set the context in which norms can be changed and stigma addressed, but they cannot affect these things in isolation. This may be an argument for focusing mass media interventions on raising awareness and knowledge and delivering them alongside other more in-depth interventions in order to enable behaviour change.

## Competing interests

PW is an author of several publications included in this review. He was not involved in the data extraction or quality assessment of any of the included studies. Other authors declare no competing interests.

## Authors’ contributions

CB, PW and KW conceived the study. RF developed and ran the search strategy, identified studies for inclusion, extracted study data and provided a narrative synthesis of the findings, which was all reviewed by CB. RF drafted the manuscript, with all authors commenting and contributing to drafts, and reading and approving the final draft.

## Pre-publication history

The pre-publication history for this paper can be accessed here:

http://www.biomedcentral.com/1471-2458/14/616/prepub

## Supplementary Material

Additional file 1 PRISMA 2009 Checklist. Click here for file

Additional file 2 Search Strategy: Ovid Medline – Total number of hits = 1315A. Click here for file
