# Supplementary material for: An exploratory review of HIV prevention mass media campaigns targeting men who have sex with men
Source: BMC Public Health. 2014 Jun 18;14:616. doi: 10.1186/1471-2458-14-616 (PMC4089926; doi:10.1186/1471-2458-14-616)
Supplement: Additional file 2 — Search Strategy: Ovid Medline – Total number of hits = 1315A. [file 1471-2458-14-616-S2.docx]

**Search Strategy: Ovid Medline – Total number of hits=1315**

This search strategy was adapted from the strategy used by Vidanapathirana et al (2005) in their systematic review of HIV testing mass media campaigns.[8] Although small media only campaigns were excluded from our review, terms relating to small media (i.e. ‘posters’ and ‘pamphlets’) were retained to increase the sensitivity of our search, so that multi-media campaigns, for example, would not be missed. Search terms relating to sexual transmitted infection were also included to ensure any relevant papers were not missed, but media campaigns only looking at STI prevention and not HIV prevention were excluded from the review. The following search terms were used to locate the relevant studies:

1. exp Homosexuality, Male/ or gay men.mp.

2. homosexual*.mp. [mp=protocol supplementary concept, rare disease supplementary concept, title, original title, abstract, name of substance word, subject heading word, unique identifier]

3. bisexual*.mp. [mp=protocol supplementary concept, rare disease supplementary concept, title, original title, abstract, name of substance word, subject heading word, unique identifier]

4. men who have sex with men.mp. or exp Bisexuality/

5. MSM.mp.

6. 1 or 2 or 3 or 4 or 5

7. media*.mp. or exp Communications Media/ or exp Mass Media/

8. communication*.mp. or exp Health Communication/

9. multimedia*.mp.

10. Video Recording/ or exp Audiovisual Aids/ or audiovisual equipment.mp. or Television/

11. patient information.mp.

12. visual information.mp.

13. radio.mp. or exp Radio/

14. television.mp. or exp Television/

15. leaflet*.mp.

16. poster*.mp. or Advertising as Topic/

17. exp Pamphlets/ or pamphlet*.mp.

18. broadcast*.mp.

19. film*.mp.

20. exp Telecommunications/ or telecommunication*.mp.

21. internet.m_titl.

22. web based.mp.

23. 7 or 8 or 9 or 10 or 11 or 12 or 13 or 14 or 15 or 16 or 17 or 18 or 19 or 20 or 21 or 22

24. HIV.mp. or exp HIV/

25. exp Sexually Transmitted Diseases/ or STI*.mp.

26. sexually transmitted infection*.mp.

27. STD*.mp.

28. sexually transmitted disease*.mp

29. 24 or 25 or 27 or 28

30. 6 and 23 and 29

31. limit 30 to yr="1990 -Current"

32. limit 31 to English language
